# Emergency department wait time in Newfoundland and Labrador, Canada: Trends and projections of physician initial assessment 2015–2026

**DOI:** 10.1371/journal.pone.0349613

**Published:** 2026-05-19

**Authors:** Moein Yoosefi, Armin Hatefi, Hensley H. Mariathas, Christopher Patey, Paul Norman, Oliver Hurley, Bahareh Ahmadzadeh, Aswathy Geetha Manukumar, Anna Walsh, Shabnam Asghari

**Affiliations:** 1 Department of Mathematics and Statistics, Memorial University of Newfoundland, St. John’s, Newfoundland, Canada; 2 Centre for Rural Health Studies, Faculty of Medicine, Memorial University of Newfoundland, St. John’s, Newfoundland, Canada; 3 Family & Emergency Medicine, Discipline of Family Medicine, Faculty of Medicine, Memorial University of Newfoundland, St. John’s, Newfoundland, Canada; 4 Carbonear Institute for Rural Reach and Innovation by the Sea, Carbonear General Hospital, Carbonear, Newfoundland, Canada; 5 Newfoundland and Labrador Health Services, Department of Quality and Learning Health Systems, Data and Information Services, St. John's, Newfoundland and Labrador, Canada; University of Michigan, UNITED STATES OF AMERICA

## Abstract

**Background:**

This study aims to analyze and project the trend of physician initial assessment (PIA) time in rural and urban emergency departments (ED’s) across Newfoundland and Labrador, Canada, considering different levels of the Canadian Triage and Acuity Scale (CTAS).

**Methods:**

The data for this study were obtained from the Newfoundland and Labrador Health Services Digital Health Department (NLHS-DH), sourced from electronic health records of emergency departments in Newfoundland and Labrador, from April 2015 to the end of 2021. A mixed-effects model was used to analyze trends in PIA time. This process also projects the mean PIA time from 2015 to 2026 across different rural/urban areas and CTAS levels (3–5). Furthermore, the Monte Carlo simulation methods are employed to estimate the uncertainty interval associated with PIA time.

**Results:**

Our projection showed that the overall mean PIA time in Newfoundland and Labrador emergency departments is expected to increase from a model-estimated 1 hour and 32 minutes in 2015 to a projected 2 hours and 59 minutes in 2026, corresponding to a 94.3% increase over the study period. This increasing trend is observed across various rural and urban areas, including varying CTAS levels. Urban regions are projected to have the biggest change, with a PC of 126.73% as compared to rural areas. The greatest overall change in PIA time is from 1 hour 25 minutes in 2015 to 3 hours 44 minutes in 2026 for CTAS 5 (PC = 163.34). However, when considering rural and urban areas separately, the most notable shift occurs for CTAS level 3 for both areas.

**Conclusion:**

PIA time trends and patterns have drastically changed across CTAS levels 3–5 in both rural and urban areas. An intervention is needed to control the increasing delay in PIA time, with a particular emphasis on urgent patients residing in rural areas.

## Introduction

### Problem

When the demand for emergency services exceeds an emergency department’s (ED) capacity to deliver quality care within appropriate time frames, it results in ED congestion [1]. There is a prominent connection between ED overcrowding and the quality of care in the ED. Patients experiencing severe pain, therefore, may not receive timely treatments or might leave the hospital without being seen [2,3]. ED overcrowding results in long wait times, which is the main cause of patient dissatisfaction [3]. Longer wait times can negatively impact the quality of patient care, satisfaction, and treatment effectiveness [4].

### Importance of predicting wait times

Given these challenges, the importance of predicting wait times becomes evident. Accurately predicting ED wait times can provide patients with valuable information regarding potential delays in receiving care, thereby increasing patient satisfaction [5]. It also enables healthcare providers and staff to better manage patient flow, improving their effectiveness and efficiency [6]. In addition to enhancing patient and staff satisfaction, the ability to predict wait times would be a practical planning tool for the government and health authorities. This would allow decision-makers to understand trends in ED wait times and adjust resource and workforce allocation accordingly. Extensive research has been conducted to predict wait times and identify factors contributing to prolonged delays across various healthcare sectors, particularly in EDs [5,7–10]. Previous studies have applied a range of approaches, including quantile regression, time-series, and more recent machine learning techniques, to forecast ED wait times [5,7–10].

### PIA definition and our goal

The purpose of the study is to predict the time it takes from a patient's arrival at the hospital to the first assessment by a physician or mid-level provider (e.g., nurse practitioner). This timeframe is known as the Physician Initial Assessment (PIA) time [11]. Timely medical attention is a key determinant of patient satisfaction in EDs, and the PIA time plays a critical role in shaping this experience. Prolonged PIA times have been associated with increased patient dissatisfaction, higher rates of patients leaving without being seen, and overall negative perceptions of care. Conversely, shorter PIA times enhance the likelihood that patients feel acknowledged and prioritized, contributing to improved satisfaction and more efficient ED operations [12,13]. In this study, the trajectory of PIA time from 2015 to 2026 is modelled using a linear mixed-effects model with spline-based time effects to capture nonlinear temporal trends while accounting for variation across healthcare facilities.

## Methods

### Study area

This study is a secondary data analysis. Data were accessed for research purposes during the period of 01/01/2024 to 31/12/2024. The data used to produce PIA time trends was obtained from five EDs in Newfoundland and Labrador’s eastern region which, three EDs were rural, and two were urban.

### NLHS-DH data

Newfoundland and Labrador Health Services’ Digital Health (NLHS-DH) Department is an institution dedicated to delivering high-quality health information to various stakeholders, including healthcare professionals, the general public, researchers, and decision-makers within the health system [14]. Patient record-level data provided by NLHS-DH for this study are extracted from Meditech, the province’s electronic health record (EHR), which collects patient-related health administrative data. This study used individual-level data that were fully de-identified before the authors accessed them. The dataset contained no personally identifiable information, such as names or unique identifiers.

### Study variables

The variables used in our time trends include the age (0–15, 16–24, 25–64, and ≥65 years), sex (female and male) of the patient, season (spring, summer, fall, winter), the time of the ED visit (12:00 AM–5:59 AM, 6:00 AM–11:59 AM, 12:00 PM–5:59 PM, and 6:00 PM–11:59 PM), where three facilities are located in rural areas, while two are in urban areas [15]. Also the CTAS level is another variable which ranks patient illness severity on a 5-point scale; CTAS level 1 represents the highest acuity and level 5 the lowest acuity [16]. The emergence of COVID-19 led to an unforeseen disruption in wait times, prompting the integration of a new variable for more accurate predictions. To account for the disruption caused by the COVID-19 pandemic, we introduced a pandemic indicator variable coded as 0 for visits in 2015–2019 and 1 for visits in 2020–2021.

The study outcome, PIA time, was calculated from the patient’s arrival time or exact time of ED visit to the first physician or mid-level provider assessment [11]. This time is computed by determining the maximum time difference between the arrival time and ED assessment, which can be identified using the patients’ registration and triage times. The first two levels of CTAS were included in the model to account for their significant impact on ED resources and overall patient flow. However, these patients’ PIA projections were not reported, as they are typically assessed immediately upon arrival due to the urgency of their condition. Consequently, variability in their wait times is minimal and less informative for trend analysis.

### Data cleaning

A systematic data cleaning and preparation process was applied to ensure the integrity and reliability of the dataset. No clinical exclusion criteria were applied beyond the data cleaning and preparation procedures. All eligible ED visits were retained in the analysis, including clinically distinct cases such as patients who were dead on arrival, died in the ED, required organ donation coordination, or underwent geriatric evaluation, where present in the dataset. Records with missing values in the outcome variable (PIA time) were excluded from the analysis. For covariates, missingness was minimal (less than 2% for most variables). We assessed whether the missing data pattern was random using a test of MCAR (Missing Completely at Random), which supported the assumption of missing observations are completely at random [17]. Accordingly, complete-case analysis was adopted rather than imputation, as the negligible proportion of missing covariate data posed minimal risk of bias. No matching methods were employed in this study. Confounding was addressed through the inclusion of relevant covariates in the statistical model.

### Statistical analysis

We estimated the mean PIA time and its associated uncertainty intervals across demographic and hospital-related factors. These factors included sex, age, year, season, CTAS level, ED location, time of ED visit, and the pandemic indicator. To do this, we used linear mixed-effects models combined with natural cubic spline regression and simulation-based analysis. [18,19]. The predictions of PIA time are estimated for interpolation and extrapolation from the first month of 2015 to the end of 2026. Initially, a mixed-effects model was utilized to investigate the influence of covariates on wait times (i.e., PIA time) and subsequently make predictions for data points of CTAS levels and rural/urban areas. The EDs were treated as a random variable in the linear mixed-effect model, while time (year) was modelled using a natural cubic spline to allow for potential nonlinear trends over time. Natural cubic splines allow flexible modelling of non-linear temporal patterns while constraining the function to become linear beyond the boundary knots. This property helps produce more stable projections when extrapolating beyond the observed data period [19,20]. Model-based predictions were used to estimate PIA time across the study period, allowing for interpolation of observed data between 2015 and 2021 and extrapolation of projected values from 2022 to 2026. The final model, which was used in this study to predict PIA from 2015 to 2026, is defined below:


$$\begin{array}{c} {PIA}_{ij}=\beta_{\text{0}}+\text{f}\left({Year}_{ij}\right)+\beta_{\text{1}}{Pandemic}_{ij}+\beta_{\text{2}}{Age}_{ij}+\beta_{\text{3}}{Season}_{ij}+\beta_{\text{4}}{ED\_Visit\_Time}_{ij}+\beta_{\text{5}}{Sex}_{ij}+\beta_{\text{6}}{CTAS}_{ij}+b_{\text{0}j}+\varepsilon_{ij} \end{array}$$

where:

• $f({\text{Year}}_{ij})$ is a natural cubic spline of the year for patient i-th and the hospital j,

• $\beta_{\text{0}}$is the fixed intercept,

• $\beta_{\text{1}},\beta_{\text{2}},\beta_{\text{3}},\beta_{\text{4}},\beta_{\text{5}},\beta_{\text{6}}$ are fixed-effect coefficients,

• $b_{\text{0}j}$is the random intercept for the hospital j,

• $\varepsilon_{ij}istheerrortermofthemodel$

After fitting the model to the observed data from 2015 to 2021, we constructed a prediction dataset containing the combinations of covariate values of interest for each year from 2015 to 2026. This prediction grid includes year, CTAS level, rural/urban setting, season, sex, age, pandemic indicator variable, and time of ED visit. The fitted model was then applied to this dataset to generate predicted mean PIA values. For the observed years, predictions represent model-based fitted values; for 2022–2026, they represent extrapolated model-based projections under the same estimated parameter structure.

The Monte Carlo algorithm is used for simulation, generating 1000 iterations and computing the associated uncertainty. From the 1,000 sets of predictions, we calculated the 2.5th and 97.5th percentiles at each time point to construct 95% uncertainty intervals [21,22]. Utilizing an uncertainty interval illustrates a conceptual range for the mean PIA time in our model. Therefore, discussing the significance of variables and whether there are overlaps within this uncertainty interval is not anticipated. To provide a practical measure of long-term change, we calculated the Percentage Change (PC) index for PIA time between 2015 and 2026. The PC was computed as:


$$\begin{array}{c} \text{PC}=\frac{{\text{PIA}}_{\text{2026}}-{\text{PIA}}_{\text{2015}}}{{\text{PIA}}_{\text{2015}}}\times \text{100}, \end{array}$$

where ${\text{PIA}}_{\text{2}\text{0}\text{1}\text{5}}$represents the predicted value of PIA in 2015, and ${\text{PIA}}_{\text{2}\text{0}\text{2}\text{6}}$represents the predicted value 2026 obtained from the fitted model [23]. All statistical analyses were performed in the R software version 4.3.2 [24]. Linear mixed-effects models were fitted using the nlme package, and temporal trends were modelled using natural splines implemented in the splines package.

### Ethics

Ethics approval for this study was obtained from the Health Research Ethics Board of Newfoundland and Labrador (HREB #2019.264). This study involved secondary analysis of fully de-identified health administrative data provided by Newfoundland and Labrador Health Services Digital Health Department. The dataset contained no personally identifiable information. As there was no direct contact with participants, informed consent was not required in accordance with institutional guidelines.

## Result

A total of 1,016,831 observations were recorded by 235,699 unique patients from April 1, 2015, to the end of December 2021 interval. After applying cleaning and preparation criteria, the dataset includes a total of 822,733 observations from 218,921 unique patients. As observed in Table 1, there are characteristics and descriptions of the dataset for a portion of covariates. The percentage of patients in the ED was higher during the summer compared to other seasons (26.1%). Approximately 57% of the records included in our dataset are attributed to the two urban hospitals in St. John’s. The proportion of patients by sex in the ED during the 2015–2021 interval was found to be 55.3% female and 44.7% male from the 2015–2021 interval. The mean age (SD) for patients was 50.9 (20) and 47.4 (24.4) years old in urban and rural areas, respectively.

**Table 1:** Demographic and clinical characteristics of rural and urban populations for real data (2015 to 2021).

Variables		Rural	Urban	Overall
**(N = 354237)**	**(N = 468496)**	**(N = 822733)**
CTAS	1	444 (0.1%)	1141 (0.2%)	1585 (0.2%)
	2	14458 (4.1%)	54579 (11.6%)	69037 (8.4%)
	3	116581 (32.9%)	216905 (46.3%)	333486 (40.5%)
	4	203688 (57.5%)	180443 (38.5%)	384131 (46.7%)
	5	19066 (5.4%)	15428 (3.3%)	34494 (4.2%)
SEX	F	197013 (55.6%)	257590 (55.0%)	454603 (55.3%)
	M	157224 (44.4%)	210906 (45.0%)	368130 (44.7%)
AGE	Mean (SD)	47.4 (24.4)	50.9 (20.0)	49.4 (22.1)
	Median [Q1, Q3]	50.5 [27.8, 67.1]	51.0 [33.3, 67.0]	50.8 [31.5, 67.0]
Age groups	0-15 Years	45707 (12.9%)	58 (0.0%)	45765 (5.6%)
	16-24 Years	33770 (9.5%)	56802 (12.1%)	90572 (11.0%)
	25-64 Years	174690 (49.3%)	280529 (59.9%)	455219 (55.3%)
	More than 65 Years	100070 (28.2%)	131107 (28.0%)	231177 (28.1%)
Season	Fall	90780 (25.6%)	122609 (26.2%)	213389 (25.9%)
	Spring	88368 (24.9%)	119622 (25.5%)	207990 (25.3%)
	Summer	92323 (26.1%)	122700 (26.2%)	215023 (26.1%)
	Winter	82766 (23.4%)	103565 (22.1%)	186331 (22.6%)
PIA	Mean (SD)	1 h 7 m (1 h 17 m)	2 h 2 m (1 h 54 m)	1 h 38 m (1 h 43 m)
	Median [Q1, Q3]	0 h 40 m (0 h 17 m, 1 h 29 m)	1 h 28 m (0 h 43 m, 2 h 45 m)	1 h 5 m (0 h 28 m, 2 h 14m)

**Fig 1:**
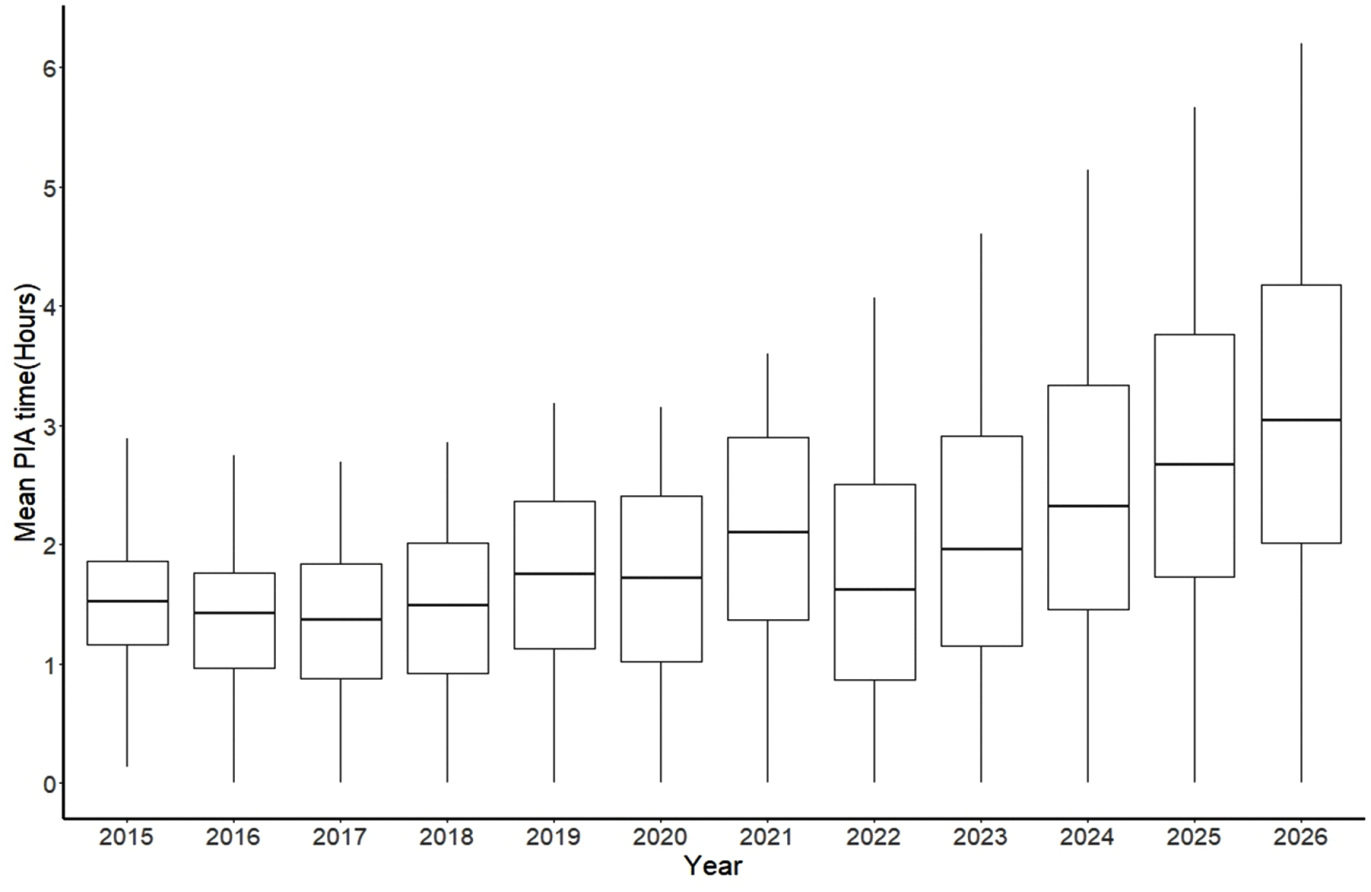
Box plot for the trend of mean PIA time over years by adjusting sex, age, year, seasons, CTAS levels, ED locations and the time of ED visit.

The mean (SD) of PIA time for the original data from 2015 to 2021 is 1h 38 m (1h 43 m), and its median [Q1, Q3] is 1h 5 m [28 m, 2h 14 m] (Table 1 and Fig 1). As shown in Fig 1, the distribution of PIA time is right-skewed across all years, with the majority of patients assessed within 1–2 hours. The observations beyond the upper whiskers represent patients who experienced extended wait times. After implementing the linear mixed model, it became evident that factors such as the patient's age, the year, CTAS levels, hospital, and time of ED visit significantly influence PIA time (P-value<0.001). We set the fall season as the reference for the seasonality variable in our model. Our findings indicate that winter is the only season not significantly different from fall (P-value = 0.57).

**Fig 2:**
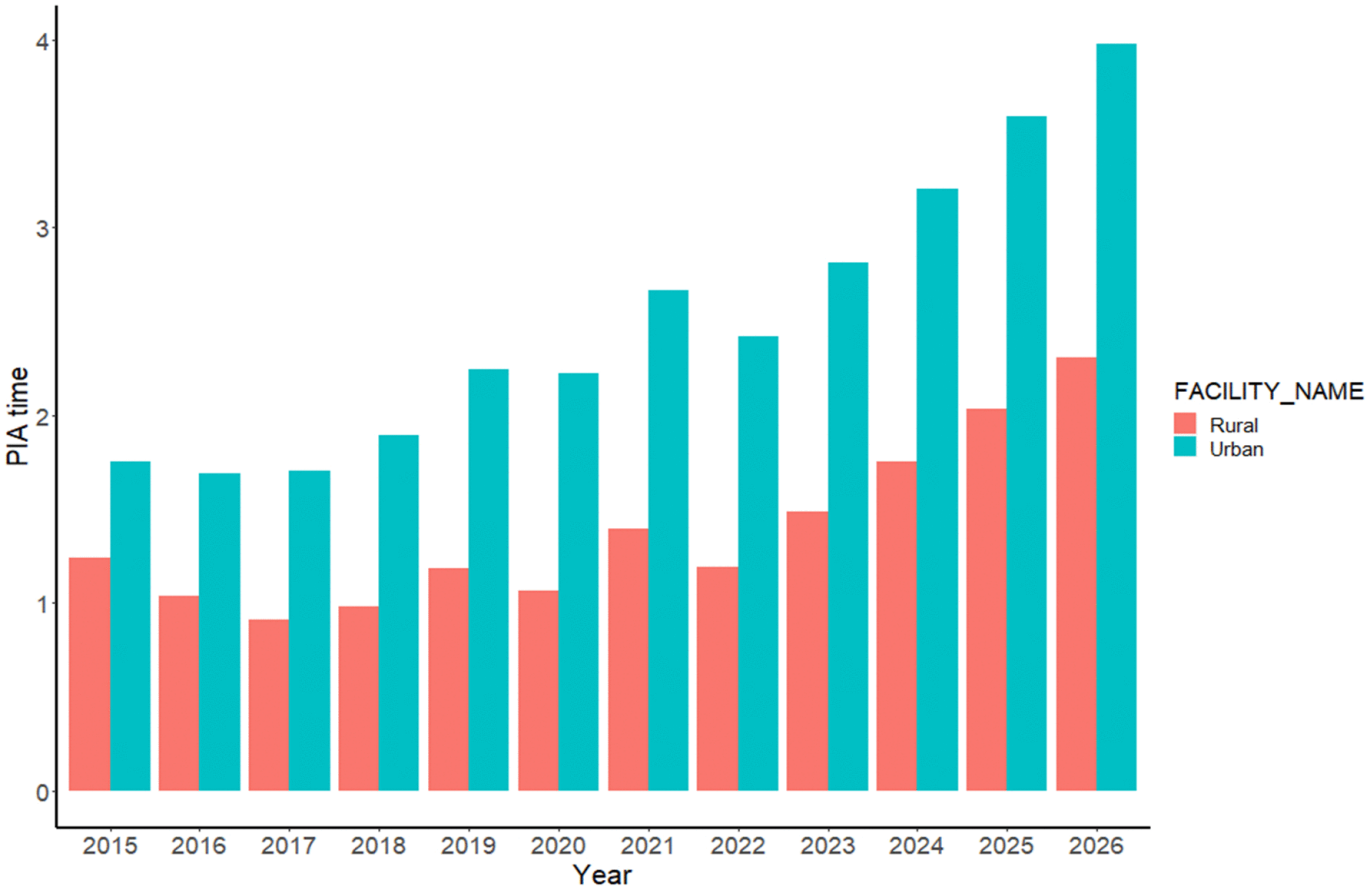
Comparison of mean PIA time in urban and rural areas over years by adjusting sex, age, year, seasons, CTAS levels, ED locations and the time of ED visit.

According to our model’s prediction for PIA time, the total mean PIA time increased from 1h 32m in 2015 to 2h 59m in 2026 (Table 2) with total percent change of 94.3%, and the median of this time is shown in Fig 1. PIA time in rural hospitals is projected to increase from 1h 15m (2015) to 2h 18m (2026), while in urban hospitals it is predicted to increase from 1h 45m in 2015 to 3h 59m in 2026 (Fig 2 and Table 2). The PC of PIA time in rural areas (85.59%) compared to urban areas (126.73%) indicates a substantial increase in PIA time in urban areas from 2015 to 2026 (Table 2, Fig 2). As illustrated in Fig 2, both rural and urban settings show the same overall temporal pattern; however, the gap between urban and rural PIA times widens progressively from 2018 onward.

**Table 2:** The mean of PIA time and percent of change by different levels of CTAS levels and rural/urban during 2015 and 2026 by adjusting sex, age, year, seasons, CTAS levels, ED locations and the time of ED visit.

Total		2015	2016	2017	2018	2019	2020	2021	2022	2023	2024	2025	2026	Percent Change (%)
1 h 32 m (0 h 39 m-2 h 25 m)	1 h 24 m (0 h 32 m-2 h 17 m)	1 h 21 m (0 h 24 m-2 h 18 m)	1 h 29 m (0 h 27 m-2 h 30 m)	1 h 45 m (0 h 35 m-2 h 55 m)	1 h 43 m (0 h 28 m-2 h 56 m)	2 h 6 m (0 h 45 m-3 h 27 m)	1 h 41 m (0 h 19 m-3 h 3 m)	2 h 1 m (0 h 40 m-3 h 23 m)	2 h 20 m (0 h 59 m-3 h 43 m)	2 h 39 m (1 h 18 m-3 h 59 m)	2 h 59 m (1 h 38 m-4 h 19 m)	94.3
**CTAS**	**3**	1 h 25 m (0 h 45 m-2 h 5 m)	1 h 20 m (0 h 35 m-2 h 5 m)	1 h 21 m (0 h 30 m-2 h 13 m)	1 h 31 m (0 h 32 m-2 h 29 m)	1 h 48 m (0 h 43 m-2 h 53 m)	1 h 45 m (0 h 32 m-2 h 55 m)	2 h 8 m (0 h 49 m-3 h 26 m)	2 h 4 m (0 h 45 m-3 h 23 m)	2 h 28 m (1 h 10 m-3 h 46 m)	2 h 50 m (1 h 32 m-4 h 8 m)	3 h 14 m (1 h 56 m-4 h 31 m)	3 h 37 m (2 h 18 m-4 h 55 m)	153.87
	**4**	1 h 47 m (0 h 58 m-2 h 36 m)	1 h 35 m (0 h 41 m-2 h 30 m)	1 h 29 m (0 h 30 m-2 h 29 m)	1 h 34 m (0 h 29 m-2 h 39 m)	1 h 51 m (0 h 37 m-3 h 4 m)	1 h 50 m (0 h 32 m-3 h 8 m)	2 h 12 m (0 h 46 m-3 h 37 m)	2 h 34 m (1 h 8 m-4 h 0 m)	3 h 1 m (1 h 36 m-4 h 27 m)	3 h 25 m (2 h 1 m-4 h 51 m)	3 h 51 m (2 h 28 m-5 h 15 m)	4 h 16 m (2 h 50 m-5 h 41 m)	139.13
	**5**	1 h 25 m (0 h 48 m-2 h 1 m)	1 h 13 m (0 h 33 m-1 h 54 m)	1 h 10 m (0 h 23 m-1 h 57 m)	1 h 21 m (0 h 28 m-2 h 14 m)	1 h 36 m (0 h 39 m-2 h 33 m)	1 h 36 m (0 h 34 m-2 h 37 m)	2 h 0 m (0 h 54 m-3 h 8 m)	2 h 10 m (1 h 2 m-3 h 16 m)	2 h 33 m (1 h 25 m-3 h 39 m)	2 h 57 m (1 h 50 m-4 h 4 m)	3 h 20 m (2 h 13 m-4 h 26 m)	3 h 44 m (2 h 39 m-4 h 49 m)	163.34
**Rural/ Urban**	**Rural**	1 h 15 m (0 h 33 m-1 h 56 m)	1 h 2 m (0 h 23 m-1 h 41 m)	0 h 55 m (0 h 14 m-1 h 34 m)	0 h 59 m (0 h 18 m-1 h 39 m)	1 h 11 m (0 h 29 m-1 h 54 m)	1 h 4 m (0 h 19 m-1 h 48 m)	1 h 24 m (0 h 37 m-2 h 12 m)	1 h 12 m (0 h 24 m-1 h 59 m)	1 h 29 m (0 h 41 m-2 h 17 m)	1 h 45 m (0 h 58 m-2 h 32 m)	2 h 2 m (1 h 15 m-2 h 49 m)	2 h 18 m (1 h 31 m-3 h 6 m)	85.59
	**Urban**	1 h 45 m (0 h 57 m-2 h 35 m)	1 h 41 m (0 h 57 m-2 h 26 m)	1 h 42 m (0 h 60 m-2 h 25 m)	1 h 54 m (1 h 8 m-2 h 39 m)	2 h 15 m (1 h 26 m-3 h 4 m)	2 h 14 m (1 h 22 m-3 h 5 m)	2 h 40 m (1 h 46 m-3 h 35 m)	2 h 25 m (1 h 31 m-3 h 19 m)	2 h 49 m (1 h 54 m-3 h 43 m)	3 h 12 m (2 h 17 m-4 h 7 m)	3 h 35 m (2 h 41 m-4 h 30 m)	3 h 59 m (3 h 4 m-4 h 53 m)	126.73

**Fig 3:**
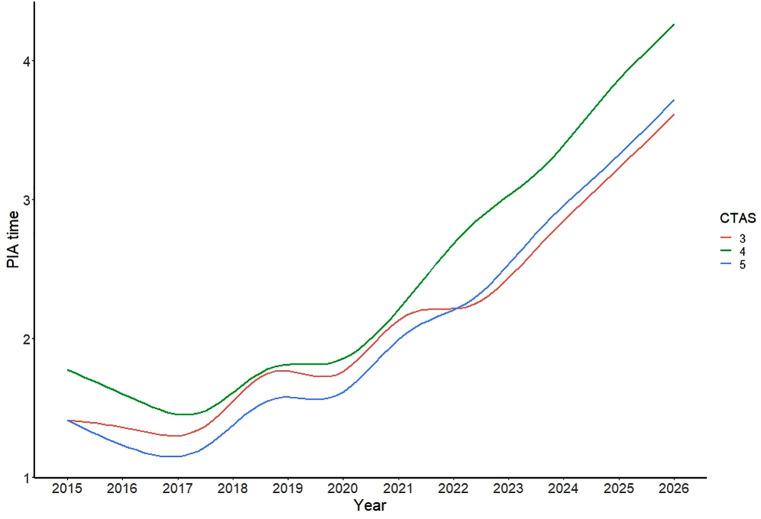
Comparison of mean PIA time in CTAS levels over years by adjusting sex, age, year, seasons, CTAS levels, ED locations and the time of ED visit.

The second-highest mean PIA time fluctuates, transitioning between CTAS 3 and CTAS 5 (Table 2). The longest PIA time is associated with level 4, increasing from 1h 47m in 2015 to 4h 16m by 2026, marking a pronounced PC of 139.13% (Fig 3, Table 2). Fig 3 shows that all three CTAS levels follow a similar J-shaped trajectory, declining from 2015 to approximately 2017 before rising sharply. In the projected period, CTAS 3 exceeds CTAS 5, reversing their earlier ordering.

The distribution of the proportion of patients in CTAS levels, as patients’ assessment levels, differs in rural and urban areas. In rural areas, the proportion of ED presentations occurs at CTAS level 4 (57.5%), while in urban areas, the highest proportion of patients is observed at CTAS level 3 (46.3%) (Table 1). Throughout 2015–2026, the most significant changes of the mean PIA is for CTAS level 3 within rural hospitals, which increased from 1h 1m to 2h 50m. Urban hospitals have experienced a similar trend, with PIA time increasing from 1h 37m in 2015 to 4h 48m by 2026 for CTAS level 3. Notably, the PC in PIA time for CTAS level 3 patients is higher in urban areas, demonstrating an increase of 196.64%, compared to the 178.79% observed in rural settings (Table 3). A detailed table summarizing the fixed- and random-effect estimates and their standard errors is provided in the Supplementary (S1 and S2 Tables).

**Table 3:** The mean of PIA time and percent of change during 2015 and 2026 by adjusting sex, age, year, seasons, CTAS levels, rural/urban, ED locations and the time of ED visit in levels of CTAS and rural/urban.

	CTAS Levels	2015	2016	2017	2018	2019	2020	2021	2022	2023	2024	2025	2026	Percent Change (%)
**Rural**	**3**	1 h 1 m (0 h 29 m-1 h 33 m)	0 h 51 m (0 h 19 m-1 h 24 m)	0 h 46 m (0 h 13 m-1 h 19 m)	0 h 51 m (0 h 17 m-1 h 25 m)	1 h 5 m (0 h 30 m-1 h 39 m)	0 h 55 m (0 h 20 m-1 h 30 m)	1 h 14 m (0 h 36 m-1 h 53 m)	1 h 29 m (0 h 44 m-2 h 13 m)	1 h 49 m (1 h 3 m-2 h 35 m)	2 h 9 m (1 h 21 m-2 h 58 m)	2 h 29 m (1 h 40 m-3 h 18 m)	2 h 50 m (1 h 59 m-3 h 41 m)	178.79
	**4**	1 h 26 m (0 h 52 m-2 h 0 m)	1 h 10 m (0 h 34 m-1 h 47 m)	1 h 1 m (0 h 22 m-1 h 41 m)	1 h 5 m (0 h 24 m-1 h 46 m)	1 h 18 m (0 h 36 m-2 h 1 m)	1 h 12 m (0 h 28 m-1 h 56 m)	1 h 32 m (0 h 46 m-2 h 18 m)	1 h 57 m (1 h 1 m-2 h 52 m)	2 h 19 m (1 h 22 m-3 h 16 m)	2 h 41 m (1 h 42 m-3 h 41 m)	3 h 5 m (2 h 3 m-4 h 6 m)	3 h 27 m (2 h 21 m-4 h 32 m)	139.77
	**5**	1 h 13 m (0 h 44 m-1 h 43 m)	0 h 59 m (0 h 31 m-1 h 28 m)	0 h 53 m (0 h 22 m-1 h 23 m)	0 h 56 m (0 h 24 m-1 h 29 m)	1 h 9 m (0 h 34 m-1 h 44 m)	1 h 3 m (0 h 24 m-1 h 41 m)	1 h 27 m (0 h 43 m-2 h 11 m)	1 h 39 m (0 h 46 m-2 h 33 m)	2 h 0 m (1 h 3 m-2 h 56 m)	2 h 20 m (1 h 21 m-3 h 18 m)	2 h 40 m (1 h 39 m-3 h 41 m)	3 h 1 m (1 h 57 m-4 h 5 m)	146.13
**Urban**	**3**	1 h 37 m (1 h 11 m-2 h 3 m)	1 h 35 m (1 h 9 m-2 h 2 m)	1 h 39 m (1 h 11 m-2 h 7 m)	1 h 53 m (1 h 23 m-2 h 22 m)	2 h 15 m (1 h 43 m-2 h 45 m)	2 h 14 m (1 h 41 m-2 h 47 m)	2 h 41 m (2 h 6 m-3 h 17 m)	2 h 59 m (2 h 15 m-3 h 43 m)	3 h 26 m (2 h 39 m-4 h 13 m)	3 h 53 m (3 h 2 m-4 h 43 m)	4 h 21 m (3 h 28 m-5 h 14 m)	4 h 48 m (3 h 52 m-5 h 44 m)	196.64
	**4**	2 h 10 m (1 h 36 m-2 h 45 m)	2 h 2 m (1 h 31 m-2 h 33 m)	2 h 0 m (1 h 32 m-2 h 29 m)	2 h 13 m (1 h 45 m-2 h 40 m)	2 h 35 m (2 h 10 m-2 h 60 m)	2 h 36 m (2 h 12 m-3 h 0 m)	3 h 5 m (2 h 42 m-3 h 28 m)	3 h 32 m (3 h 1 m-4 h 3 m)	4 h 2 m (3 h 29 m-4 h 34 m)	4 h 31 m (3 h 59 m-5 h 4 m)	5 h 1 m (4 h 27 m-5 h 34 m)	5 h 30 m (4 h 55 m-6 h 6 m)	153.74
	**5**	1 h 42 m (1 h 15 m-2 h 9 m)	1 h 37 m (1 h 10 m-2 h 3 m)	1 h 37 m (1 h 10 m-2 h 5 m)	1 h 47 m (1 h 17 m-2 h 17 m)	2 h 6 m (1 h 34 m-2 h 39 m)	2 h 3 m (1 h 29 m-2 h 37 m)	2 h 30 m (1 h 52 m-3 h 9 m)	2 h 57 m (2 h 11 m-3 h 44 m)	3 h 24 m (2 h 34 m-4 h 13 m)	3 h 52 m (2 h 59 m-4 h 44 m)	4 h 19 m (3 h 23 m-5 h 14 m)	4 h 46 m (3 h 47 m-5 h 45 m)	181.01

## Discussion

This study aims to identify trends and projections in PIA times in Newfoundland and Labrador, Canada, from 2015 to 2026. The findings indicate an upward trend in predicting the mean of PIA time over total time and different levels of CTAS (3–5) and rural/urban areas. If decision makers do not intervene to control ED wait times, the mean PIA time will increase to 2h 59m in 2026.

### Mean and trend of PIA

Based on existing data from NLHS-DH, the median PIA time from April 2015 to December 2021 is 1h 5m. The wait time in an Alberta ED between April 2010 and March 2015 was 1h 23m, while the time for an Ontario ED from April 2010 to March 2015 was 60 minutes [1,25].

Our study showed that the trend of PIA time is increasing from 2015 to 2026 in Newfoundland and Labrador. The total PC over this period is 94.3%. Dividing this total change by the 12-year study period yields an average annual percent change (AAPC) of 7.86%. In a retrospective cohort study in Alberta, Canada, PIA times were compared across 16 high-volume regional, urban, and academic EDs from April 2010 to March 2015. That study highlighted two indices from the Canadian Association of Emergency Physicians (CAEP) recommendations for PIA times: one for PIA exceeding one hour and another for PIA exceeding three hours. For both indices, the PIA times have been increasing over the years [1]. The Canadian Institute for Health Information (CIHI) compared the mean of PIA times, specifically the 90th percentile (90% spent less time, in hours), across various provinces in Canada from 2018 to 2023. CIHI research indicated an overall increasing trend in PIA times during this period, with notable increases observed in larger provinces like British Columbia and Ontario. Their results and trends were calculated over a 5-year period from 2018–2019–2022–2023. In Ontario, PIA time increased from 3.2 to 4.2 hours over the study period, reflecting an AAPC of 6.25%. Similarly, in British Columbia, this time went up from 3.1 to 4.0 hours, showing an AAPC of 5.8% [26,27]. This demonstrates that the AAPC for a variety of provinces are similar.

### The trend of PIA in rural and urban areas

Patients in rural and urban areas may experience different waiting times for various reasons. Historically, rural EDs process fewer patients than their urban counterparts. Furthermore, due to their relatively small size, rural EDs may have other efficiencies in their processes and less administrative overhead, which could lead to faster evaluations [16]. The results of our study show an increasing trend in PIA times in both urban and rural EDs. However, the PC change in PIA time for urban areas is more than in rural areas (126.73% compared to 85.59%, respectively). Although urban areas typically benefit from more advanced healthcare infrastructure, better resource allocation, and higher staffing levels [28,29], they also face the challenge of higher patient demand and greater population density compared to rural areas [1,30]. A significant contributor to ED overcrowding in urban centers is inpatient bed boarding, where admitted patients remain in the ED due to the unavailability of inpatient beds. This situation hampers the ED's capacity to assess and treat new patients, leading to increased wait times and potential delays in care [31]. Additionally, the closure of rural EDs and the diversion of ambulances to urban hospitals exacerbate the strain on urban EDs, further intensifying overcrowding issues [32]. Staffing shortages, particularly in nursing and specialized medical roles, compound these challenges, making it difficult to maintain efficient patient flow and timely assessments [33].

### Trend of PIA by CTAS

According to the definition of CTAS levels, the priority of patient visits by physicians follows the order of the CTAS levels [16] The mean PIA times for certain years show distinct patterns across CTAS levels three to five. Typically, CTAS 4 has the highest PIA time, followed by CTAS 5. In the projected years, however, the order between CTAS 3 and CTAS 5 is reversed. The results of previous studies align with our final findings for different CTAS levels. A study examined patients over 18 years of age who visited selected EDs in Ontario between January 2016 and December 2017, finding the highest PIA time for CTAS level 3, following level 2 [34]. Another study observed the highest median PIA time for CTAS levels 3 and 4 [1]. The process can be attributed to several factors, including the availability of doctors and nurses, beds and boarding access blockages [31,35], ambulance delays, number of beds, and test processing speed [36,37]. A retrospective archive study was conducted in a tertiary hospital in northern Israel from January 2011 to December 2015. The study found that the trend of median PIA time for CTAS levels 1 and 2 remained uniform, while the median PIA time for CTAS levels 3, 4, and 5 showed an increasing trend [38].

### Trend of mean of PIA by urban ED and CTAS

The mean of PIA time trends across different CTAS levels are increasing in both rural and urban areas. Overall, the PC in urban areas from 2015 to 2026 is higher than in rural areas across all CTAS levels. Among the levels, CTAS 3 shows the most significant change over this period, with a percentage increase of 178.79% in rural areas and 196.64% in urban areas. Due to a shortage of physicians in the community in recent years, patients increasingly relied on EDs for necessary care. For instance, in our dataset, patient volume increased from 112,513 records in 2015–138,621 records in 2021. During the same period, however, the number of ED physicians and available resources did not keep pace with this growth [39].

### Baseline impact of COVID-19 in 2020

The arrival of COVID-19 changed how healthcare was delivered and affected ED wait times. When COVID-19 began in 2020, wait times decreased for several reasons. First, patients were afraid to go to hospitals for non-urgent issues due to fear of COVID-19 exposure, leading to a substantial reduction in ED visits for less acute conditions. Second, the closure of walk-in clinics and other in-person assessment sites removed alternative settings that generated referrals or redirected patients to the ED, which reduced total patient volumes [40]. Third, hospitals and clinics had to change adjust operations in response to the COVID-19 how they did things because of COVID-19. They had to rearrange and reorganize spaces, enhance infection prevention protocols (e.g., additional time associated with donning and doffing personal protective equipment), exercise greater caution in preventing infections, and modify the approach to resource redistribution.

### Limitations and merits of the study

Although median wait times are commonly reported in the ED literature due to the skewed nature of wait-time distributions, the present study used mean PIA time as the primary outcome for several reasons. First, the study objective was to model temporal trends and generate long-term projections using a linear mixed-effects framework, which estimates conditional means as its natural estimation. Second, the mean captures the influence of extreme wait times, which are operationally important for understanding the full burden of system pressures and informing resource planning. Third, comparison of mean and median PIA trends across the study period showed highly consistent trajectories across all CTAS levels and rural/urban settings, with no clinically meaningful differences in the direction or magnitude of change, confirming that conclusions drawn from the mean are generalizable to the overall distribution. Furthermore, within a parametric modelling framework, the mean provides more statistically efficient estimates than the median [19,41].

In linear mixed-effects models, the normality assumption applies to the residuals and random effects rather than to the raw outcome variable. Residual diagnostics confirmed no severe departures from normality, and with a large sample size of observations, the Central Limit Theorem ensures the asymptotic validity of parameter estimates under moderate departures. Log-transformation of PIA time was explored during model development, but did not improve projection performance and reduced clinical interpretability. The final model was therefore fitted on the original PIA time scale.

In 2020, the COVID-19 pandemic changed the pattern of PIA times compared to previous years. To ensure our model's reliability, we added a variable as a pandemic indicator variable to the data for this year. Our dataset presented several challenges, including missing data, sparsity in certain variable levels, and the presence of outliers. To address these issues, we employed advanced data wrangling and cleaning techniques and statistical methodologies, ensuring the integrity and reliability of our analysis.

The omission of separate projected results for CTAS levels 1 and 2 should not be interpreted as exclusion from the analysis. These levels were included in model fitting, but their projections were not reported because their PIA times are clinically expected to be near-immediate and therefore offer limited additional insight into temporal variation in wait times. By focusing on CTAS levels 3–5, where the variability in wait times is most pronounced, our projections offer a more accurate depiction of potential systemic challenges and inform better-targeted interventions to address delays in less acute but still significant cases. Future research could explore system efficiencies in the management of CTAS 1 and 2 to ensure resource readiness but remains beyond the scope of this trend-focused analysis.

We acknowledge that additional covariates, such as overall ED patient volume and day of the week, may influence PIA time. Both were explored during model development but were not considered in the final model. ED patient volume was highly correlated with the time of ED visit variable and the facility-level random effects already in the model, and its inclusion did not produce clinically meaningful changes in the estimates.

Day of the week was excluded from the model to avoid overparameterization. Including this variable would have added seven additional levels, which could have created sparse covariate combinations, especially in smaller rural EDs and less common CTAS levels. This sparsity may have led to unstable projections. In addition, much of the day-to-day variation was already captured by the time-of-ED-visit intervals and seasonal terms.

Future studies with larger datasets and access to real-time ED occupancy data may be better positioned to incorporate these variables and further refine PIA projections.

PIA, one of the key performance indicators for EDs, was examined in this study, and its trend was projected through the end of 2026. However, waiting time measured by length of stay, as well as the proportion of patients who left the ED without being seen by a physician, are two additional important KPIs whose trends could also be projected and analyzed. Due to differences in model structure and covariate associations, these outcomes require separate analytical approaches and will be examined in future studies.

Statistical and machine learning models have been widely used to predict ED wait times and patient volumes, helping hospitals allocate resources, improve patient flow, and reduce delays. These models provide a foundation for AI applications, which can automate triage, rapidly analyze patient information, and identify inefficiencies. However, many existing approaches focus on short-term, patient-level predictions, are difficult to interpret, and often ignore the hierarchical structure of ED data, limiting generalizability. To address these limitations, we use a spline-based linear mixed-effects model to capture temporal trends in PIA time while accounting for both patient and facility-level variability, improving operational efficiency and patient care [5,7–10].

The current model does not incorporate an explicit autoregressive correlation structure to account for temporal dependence between consecutive observations. Although this approach was explored, it was not feasible due to computational and convergence challenges associated with the size and complexity of the dataset. The spline-based time trend and facility-level random effects used in the final model provide a flexible framework for capturing the primary temporal patterns, consistent with the study's objective of estimating overall trends and between-facility variability. Future studies may explore alternative approaches, such as spatio-temporal modelling frameworks, to more explicitly account for temporal dependence in large-scale administrative health datasets.

Despite these limitations, this study makes an important contribution to the literature by establishing the baseline and projected trajectory of PIA time in Newfoundland and Labrador under current conditions. This type of evidence is essential for decision-makers because intervention planning in emergency department systems must be guided by an understanding of where the system currently stands and what future pressures are likely in the absence of change. The projections presented here can help inform the timing, scale, and targeting of future operational interventions. In addition, the modelling framework offers a reproducible basis for future updates and ongoing monitoring of ED performance trends.

### Conclusion and future plan

Our projection showed that PIA times without of intervention will continue to increase as we approach 2026, with different rates of change across rural, urban, and CTAS levels. Our projections also suggest the requirement of an intervention to manage the anticipated increases in PIA times effectively, ensuring that EDs can maintain high standards of patient care and operational efficiency amidst changing demand patterns.

Moreover, the total PIA time is expected to almost double from 2015 to 2026. This trend indicates a pressing need for targeted interventions to manage and improve healthcare wait times. Deciosion-makers should focus on improving the efficiency of urban ED healthcare by implementing targeted services and optimizing budget use. This approach ensures resources are allocated effectively to provide high-quality care while minimizing waste. These interventions could help mitigate the rising trend of PIA times and improve the wait time in Newfoundland and Labrador, ensuring more timely and equitable access to healthcare services.

## Supporting information

S1 Table Random-effect variance components of the linear mixed-effects model for PIA time (hours), including a random intercept for each ED facility (n = 5). (DOCX)

S2 Table Fixed-effect estimates of the linear mixed-effects model for PIA time (hours). Reference categories: Female, CTAS 1, Pre-pandemic (2015–2019), age < 16, Fall, and 12:00 AM–5:59 AM. ** p < 0.001; ** p < 0.01; * p < 0.05.
(DOCX)
