# Supplementary material for: Emergency department wait time in Newfoundland and Labrador, Canada: Trends and projections of physician initial assessment 2015–2026
Source: PLoS One. 2026 May 19;21(5):e0349613. doi: 10.1371/journal.pone.0349613 (PMC13186388; doi:10.1371/journal.pone.0349613)
Supplement: S1 Table — (DOCX) [file pone.0349613.s001.docx]

| Random Effect | Group | Std. Deviation |
| --- | --- | --- |
| Intercept | EDs (n = 5) | 0.6179 |
| Residual | — | 1.5429 |
