# Supplementary material for: Emergency department wait time in Newfoundland and Labrador, Canada: Trends and projections of physician initial assessment 2015–2026
Source: PLoS One. 2026 May 19;21(5):e0349613. doi: 10.1371/journal.pone.0349613 (PMC13186388; doi:10.1371/journal.pone.0349613)
Supplement: S2 Table — Reference categories: Female, CTAS 1, Pre-pandemic (2015–2019), age < 16, Fall, and 12:00 AM–5:59 AM. ** p < 0.001; ** p < 0.01; * p < 0.05. (DOCX) [file pone.0349613.s002.docx]

| Variable | | Estimate | Std. Error | p-value |
| --- | --- | --- | --- | --- |
| Intercept |  | -0.4405 | 0.2801 | 0.1159 |
| Year | Spline 1 | 0.3071 | 0.0071 | <0.001***** |
|  | Spline 2 | 0.5263 | 0.0142 | <0.001***** |
|  | Spline 3 | 1.2234 | 0.0132 | <0.001***** |
| Sex | Female |  |  |  |
|  | Male | -0.0657 | 0.0035 | <0.001***** |
| CTAS | 1 |  |  |  |
|  | 2 | 0.5157 | 0.0449 | <0.001***** |
|  | 3 | 1.4402 | 0.0445 | <0.001***** |
|  | 4 | 1.8664 | 0.0445 | <0.001***** |
|  | 5 | 1.6412 | 0.0453 | <0.001***** |
| Pandemic Indicator | 0 (2015 – 2019) |  |  |  |
|  | 1 (2020 – 2021) | -0.4430 | 0.0103 | <0.001***** |
| Age | <16 |  |  |  |
|  | 16–24 | 0.1235 | 0.0094 | <0.001***** |
|  | 25–64 | 0.1394 | 0.0081 | <0.001***** |
|  | ≥65 | 0.0885 | 0.0084 | <0.001***** |
| Season | Fall |  |  |  |
|  | Spring | -0.0825 | 0.0049 | <0.001***** |
|  | Summer | 0.0527 | 0.0049 | <0.001***** |
|  | Winter | 0.0029 | 0.0051 | 0.5698 |
| Time of ED Visit | 12:00 AM–5:59 AM |  |  |  |
|  | 6:00 AM–11:59 AM | -0.2438 | 0.0070 | <0.001***** |
|  | 12:00 PM–5:59 PM | 0.4022 | 0.0070 | <0.001***** |
|  | 6:00 PM–11:59 PM | 0.3672 | 0.0075 | <0.001***** |
